# The Role of Pharmacogenetics in Personalizing the Antidepressant and Anxiolytic Therapy

**DOI:** 10.3390/genes14051095

**Published:** 2023-05-16

**Authors:** Milica Radosavljevic, Dubravka Svob Strac, Jasna Jancic, Janko Samardzic

**Affiliations:** 1 Institute of Pharmacology, Clinical Pharmacology and Toxicology, Faculty of Medicine, University of Belgrade, 11000 Belgrade, Serbia; milica.radosavljevic.bg@gmail.com; 2 Laboratory for Molecular Neuropsychiatry, Division of Molecular Medicine, Rudjer Boskovic Institute, 10000 Zagreb, Croatia; dsvob@irb.hr; 3 Clinic of Neurology and Psychiatry for Children and Youth, Faculty of Medicine, University of Belgrade, 11000 Belgrade, Serbia; jasna.jancic.npk@gmail.com

**Keywords:** pharmacogenetics, pharmacogenomics, anxiety, depression, genetic variations, stress

## Abstract

Pharmacotherapy for neuropsychiatric disorders, such as anxiety and depression, has been characterized by significant inter-individual variability in drug response and the development of side effects. Pharmacogenetics, as a key part of personalized medicine, aims to optimize therapy according to a patient’s individual genetic signature by targeting genetic variations involved in pharmacokinetic or pharmacodynamic processes. Pharmacokinetic variability refers to variations in a drug’s absorption, distribution, metabolism, and elimination, whereas pharmacodynamic variability results from variable interactions of an active drug with its target molecules. Pharmacogenetic research on depression and anxiety has focused on genetic polymorphisms affecting metabolizing cytochrome P450 (CYP) and uridine 5’-diphospho-glucuronosyltransferase (UGT) enzymes, P-glycoprotein ATP-binding cassette (ABC) transporters, and monoamine and γ-aminobutyric acid (GABA) metabolic enzymes, transporters, and receptors. Recent pharmacogenetic studies have revealed that more efficient and safer treatments with antidepressants and anxiolytics could be achieved through genotype-guided decisions. However, because pharmacogenetics cannot explain all observed heritable variations in drug response, an emerging field of pharmacoepigenetics investigates how epigenetic mechanisms, which modify gene expression without altering the genetic code, might influence individual responses to drugs. By understanding the epi(genetic) variability of a patient’s response to pharmacotherapy, clinicians could select more effective drugs while minimizing the likelihood of adverse reactions and therefore improve the quality of treatment.

## 1. Introduction

**Figure 1:**
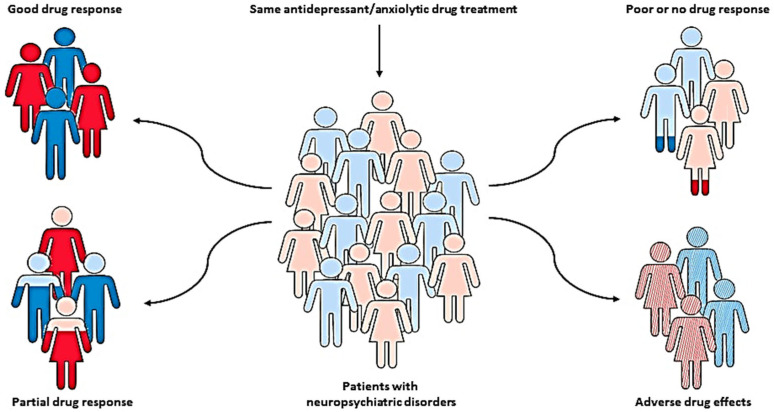
Pharmacogenetics, as a key part of personalized medicine, can help clinicians predict the therapeutic response and adverse drug reactions in patients with the same diagnosis and treatment, but different genotypes, and therefore identify patients who may optimally benefit from specific, individually tailored treatment.

The pharmacotherapy of neuropsychiatric disorders, such as anxiety and depression, has been characterized by significant inter-individual variability in drug response and the development of severe adverse effects, which has been recognized as a major clinical problem [1,2]. In addition to various environmental, physiological, and psychological factors, these individual differences might be largely due to genetic factors [3,4]. Therefore, various pharmacogenetic studies have been conducted to identify genetic variants that can predict patients who may optimally benefit from specific, individually tailored treatments [5,6,7,8,9] (Figure 1).

Pharmacogenetic studies have focused primarily on candidate genes involved in drug metabolism and transport (pharmacokinetics) as well as drug action (pharmacodynamics), which can influence both treatment efficacy and the development of adverse drug effects [9,10,11]. Pharmacokinetics addresses the variability in the drug’s absorption, distribution, metabolism, and elimination (ADME), which modulates the delivery of drugs and their active metabolites or their removal from action targets.

The molecules involved in ADME processes include enzymes responsible for drug metabolism and drug transport molecules that mediate drug uptake and efflux [4]. In this context, the cytochrome P450 (CYP) and multidrug resistance (MDR) gene families have been extensively studied [6,12]. The CYP450 enzyme family in the liver is responsible for the metabolism of many psychotropic drugs [13]. For certain CYPs, the genotype affects the serum/plasma drug levels, and consequently, its efficacy and development of adverse effects [14,15]. There are four major CYP phenotypes produced by combinations of various alleles with different degrees of enzymatic activity: poor (PM), intermediate (IM), extensive (normal) (EM), and ultrarapid metabolizer (UM). PMs tend to accumulate higher drug levels in the blood and may require lower drug doses to achieve therapeutic effects, whereas UMs may require higher doses due to faster drug elimination [16,17].

In addition, the therapeutic action of psychotropic drugs depends on their effective delivery to the brain. Although some substances may diffuse passively through the brain-blood barrier (BBB), the influx and efflux of most substances are actively regulated by a complex system of transporters, influencing both pharmacokinetics and pharmacodynamics. In the case of a genetically determined decrease in functional activity or expression of transport proteins in the BBB, drug efflux from the brain into the blood is disturbed. This could lead to increased drug exposure time in the brain, its accumulation during long-term therapy, and an increased risk of developing severe adverse effects [18].

In contrast to pharmacokinetics, pharmacodynamics describes variability in drug action not dependent on variable drug concentrations but rather on the interaction of the active drug with its target molecules, including receptors, ion channels, and enzymes, and it can also influence both therapeutic responses and drug side effects.

Single nucleotide polymorphisms (SNPs) are the most commonly investigated genetic variants in both pharmacokinetic and pharmacodynamic studies [11]. Genetic polymorphism refers to the occurrence of two or more common variants (alleles) of a specific DNA sequence in a population with a frequency of more than 1% [19]. Identifying SNPs associated with variability in drug response and toxicity has been the primary focus of a significant number of pharmacogenetic and pharmacogenomic studies [7,20,21]. In these studies, two major research approaches have been used: the traditional candidate gene approach, which is hypothesis-driven and based on accumulated knowledge, and new methodologies such as genome-wide association studies (GWAS) or whole exome sequencing, which are data-driven and generate new hypotheses and knowledge [22,23].

However, pharmacogenetics is not able to explain all observed heritable variations in drug responses, and there is growing evidence that responses to drugs could be influenced by individual epigenetic states [24,25]. An emerging field of pharmacoepigenetics investigates how epigenetic mechanisms that modify gene expression without altering the genetic code might influence individual responses to drugs [24,26]. Some of the most frequently studied epigenetic modifications include DNA methylation, histone modifications, and noncoding RNA actions [25]. Various environmental factors, including drugs, nutrition, and stress, may induce epigenetic changes [27] that can be transmitted over generations [28]. Acute or chronic exposure to stressors can contribute to the development and progression of various neuropsychiatric disorders [29,30,31,32], including anxiety and depression, but it is also associated with alterations in the epigenome that may affect the expression of genes involved in drug metabolism, transport, and target molecules, and therefore impact the variability in antidepressant and anxiolytic drug responses [33,34]. The most significant findings, obtained by both pharmacogenetic and pharmacoepigenetic research on antidepressants and anxiolytics, drugs commonly used for the therapy of various neuropsychiatric disorders, have been summarized and discussed in this review.

## 2. Pharmacogenetics of Antidepressants

Antidepressants are drugs commonly used to treat depression and anxiety disorders, although only half of patients respond to treatment, and only a third of patients experience symptom remission [35]. Genetic factors seem to account for more than 60% of the variability in drug response and side effects for various types of antidepressant drugs, including selective serotonin reuptake inhibitors (SSRIs), serotonin-norepinephrine reuptake inhibitors (SNRIs), tricyclic (TCAs) and tetracyclic compounds, monoamine oxidase inhibitors (MAOIs), and noradrenergic and serotonergic modulators [36]. Therefore, an individual’s genetic profile should be considered when choosing an antidepressant and determining the appropriate drug dosage. However, despite ongoing research, the effects of genetic variants on the pharmacokinetics and pharmacodynamics of antidepressant drugs remain unclear. Discovering the genetic factors that contribute to the variability of antidepressant responses can help clinicians select the most appropriate medication, dosage, and treatment duration for each individual patient. Therefore, the application of precision medicine may improve the treatment response rates to antidepressants and minimize the risk of adverse drug reactions.

### 2.1. Pharmacokinetic Variability

#### 2.1.1. Cytochrome P450 Family

The CYP450 family is a large group of enzymes responsible for the metabolism of various drugs and xenobiotics, including antidepressant drugs. Among the many identified CYP450 enzymes, the most important ones involved in the metabolism of a variety of psychotropic drugs are CYP2D6, CYP2C19, CYP3A4, CYP1A2, CYP2B6, CYP2C8, and CYP2C9 [9,37]. Various CYP450 enzymes are capable of metabolizing more than one drug, and a single drug can be metabolized by multiple CYP enzymes [15]. The activity of these enzymes could be influenced by genetic variations, which may result in individual differences in antidepressant drug metabolism and responses [15,16]. Most genes encoding CYPs are highly polymorphic [19]. The impact of *CYP* polymorphisms on drug metabolism is an important area of research in personalized medicine [19]. While more than 2000 mutations have been found in *CYP* genes, only specific SNPs are known to affect CYP enzymatic activity [14,15]. Depending on the genetic variants that influence enzyme activity, individuals can be classified into four main CYP phenotypes: from poor, via intermediate and extensive (normal), to ultrarapid metabolizers [17,38,39]. Due to genetic variations, PMs have little to no CYP enzyme activity, whereas IMs have reduced enzyme activity. As a result of slower antidepressant drug metabolism, both PMs and IMs may be at an increased risk for adverse drug reactions and require lower dosages or less frequent dosing regimens for antidepressant medications. On the other hand, NMs possess typical enzyme activity and a normal drug metabolism rate. However, UMs exhibit increased enzymatic activity, resulting in faster drug metabolism. Therefore, they may require higher antidepressant doses or more frequent drug administration to achieve the desired therapeutic effect in patients with depression [17,18,38,39]. The pharmacogenetics of the most important CYPs involved in the metabolism of antidepressant drugs are summarized below.

##### CYP2D6

The *CYP2D6* gene is highly polymorphic, meaning that it harbors genetic variations that can affect enzyme function [15,16,40]. CYP2D6 enzyme is involved in the metabolism of SSRIs (paroxetine, fluvoxamine, and fluoxetine), amitriptyline (TCA), and venlafaxine (SNRI) [16]. Different allele variants result in normal, decreased, and no enzyme function, characterized by extensive, intermediate, and poor metabolizer phenotypes, respectively [16,41]. CYP2D6*1, *2, *33, and *35 allele variants are associated with normal function, whereas *9, *10, *14B, *17, *29, and *41 variants reduce CYP2D6 enzymatic capacity. Furthermore, CYP2D6*3, *4, *5, *6, *7, *11, *12, *14A, *36, and *68 variants are responsible for the loss of enzyme function [16,41]. Apart from genetic variations, paroxetine and fluoxetine are potent inhibitors of CYP2D6, which may result in an “iatrogenic poor phenotype” or “phenocopy” when taken with drugs that are also metabolized by CYP2D6, such as venlafaxine. Patients with an “iatrogenic poor phenotype” may be at high risk of developing toxicity due to elevated plasma drug levels [42]. Specifically, individuals with poor CYP2D6 function may have reduced metabolism of fluoxetine and paroxetine, resulting in higher plasma drug levels and an increased risk of side effects, such as the development of suicidal ideation or antidepressant-induced mania during the initiation of treatment. As a result, the U.S. Food and Drug Administration (FDA) has issued a black box warning for SSRIs cautioning that these side effects can occur, especially in the first few weeks of treatment. Therefore, close monitoring is required for all patients starting antidepressant therapy [42,43].

Furthermore, studies have shown that poor metabolizers of fluoxetine may be at a risk of QT interval prolongation. [42]. Similar to fluoxetine, poor metabolizers of venlafaxine (genotype CYP2D6 *6/*4, *5/*4, or *6/*6) are also at risk for developing adverse effects, such as arrhythmias, gastrointestinal problems, and hyponatremia [44,45]. Ahmed et al. showed that CYP2D6 UM status contributes to venlafaxine treatment remission in patients with major depressive disorder [46]. Therefore, when prescribing SSRIs and other antidepressants, clinicians must consider a patient’s *CYP2D6* genotype and adjust the dose or choose an alternative medication, if necessary, to minimize the risk of adverse effects while maximizing therapeutic benefit [6,7,20]. Despite the growing literature on the clinical implications of the *CYP2D6* genotype and phenoconversion on patient-related outcomes, implementation of pharmacogenetics to guide antidepressant prescribing is rare.

##### CYP2C19

In addition to clinically significant variants of the *CYP2D6* gene, a high number of polymorphisms in the *CYP2C19* gene have also been discovered [47], resulting in different metabolizer phenotypes [16,41]. Drugs that are mainly metabolized by CYP2C19 include SSRIs such as escitalopram, citalopram, and sertraline [15]. Variations in the *CYP2C19* gene can result in individuals having different levels of CYP2C19 enzyme activity, affecting how drugs such as escitalopram and sertraline are metabolized [48,49]. According to previous research, the CYP2C19*1 allele variant is associated with normal function, whereas the CYP2C19*17 variant increases CYP2C19 enzymatic capacity. Furthermore, CYP2C19*2 and *3 variants are responsible for the loss of enzyme function [16,41].

For example, PMs of escitalopram are more likely to have adverse effects and psychotherapy discontinuation. However, they respond better to escitalopram, provided the therapy is tolerated. A retrospective study involving more than 2000 escitalopram-treated patients demonstrated that those with CYP2C19*1/*17 and CYP2C19*17/*17 variants (IMs) were more likely to experience treatment failure [50], whereas UMs exhibited suicidal thoughts [51]. For sertraline, recent findings demonstrated that CYP2C19 PMs had higher plasma levels in comparison to normal metabolizers [52]. Ricardo-Silgado et al. [53] suggested that *CYP2C19* genotypes might be associated with an increased risk of weight gain in patients on citalopram therapy. These findings indicate the importance of *CYP2C19* variants and different metabolic phenotypes in antidepressant treatment tolerability and outcome. The American Molecular Pathology published guidelines for testing CYP2C19*2, *3, and *17 variants and CYP2C19*4A-*4B, *5, *6, *7, *8, *9, *10, and *35 variants [41]. In addition, the relevance of *CYP2C19* polymorphisms in inter-individual predisposition to mental diseases was also investigated. Sim et al. [54] found that individuals who were poor CYP2C19 metabolizers displayed lower levels of depressive symptoms compared to individuals who were normal metabolizers. These results raise the possibility that *CYP2C19* variations contribute to susceptibility to depression. Recent studies have also demonstrated a correlation between low CYP2C19 activity and the severity of depression symptoms [55,56].

Moreover, a cross-sectional observational retrospective study, which included over 700 psychiatric patients with depression and anxiety, analyzed the frequencies of CYPC19*2, *4, and *17, as well as CYP2D6*2, *3, *4, *5, *6, *10, and *41 variants [37]. This study revealed that 77% of patients have at least one allele variant significantly affecting drug metabolism, with roughly half of the individuals with reduced CYP2D6 enzyme function and the majority of them being CYP2C19 rapid and ultrarapid metabolizers [37]. Therefore, due to their clinical relevance, both *CYP2D6* and *CYP2C19* are included in pharmacogenetics guidelines and recommendations published by the Clinical Pharmacogenetics Implementation Consortium (CPIC), Dutch Pharmacogenetics Working Group (DPWG), and regulatory agencies such as the FDA [57,58,59].

##### CYP2C9

The *CYP2C9* gene encodes an enzyme that is involved in the metabolism of many drugs, including antidepressants, such as MAOIs, TCAs, and SSRIs [17]. The CYP2C9*1 variant is associated with normal enzyme function, whereas CYP2C9*2, *5, *8, and *11 variants reduce CYP2C9 enzymatic capacity. Furthermore, CYP2D9*3, *6, and *13 variants are responsible for the loss of enzyme function [16,41]. Different *CYP2C9* variants result in normal, decreased, and no enzyme function, characterized by extensive, intermediate, and poor metabolizer phenotypes, respectively [16,41]. Moreover, variants of the *CYP2C9* gene have been linked to mental disorders such as depression and psychosis [60,61].

##### CYP1A2

The CYP1A2 enzyme, encoded by the *CYP1A2* gene, is responsible for the metabolism of approximately 24% of antidepressant drugs, including agomelatine, escitalopram, venlafaxine, duloxetine, and mirtazapine [16,36]. Several *CYP1A2* variants, including *1C, *1F, and *1B, have been identified as potential indicators of the CYP1A2 phenotype and agomelatine pharmacokinetics. Notably, the CYP1A2*1C variant is associated with reduced enzyme activity, resulting in higher plasma agomelatine concentrations and an increased risk of adverse effects. On the contrary, individuals with the CYP1A2 *1F and *1B variants, who are characterized by increased enzyme activity, may metabolize agomelatine more rapidly, leading to lower plasma concentrations and potentially reduced efficacy [62,63]. Kuo et al. [64] conducted a study to investigate the association between *CYP1A2* polymorphisms and the metabolism of escitalopram in patients with major depressive disorder. The study found that several SNPs in the *CYP1A2* gene, such as rs2069521, rs4646425, and rs4646427 polymorphisms, were associated with altered escitalopram metabolism and an increased risk of adverse effects, such as fatigue and nausea/vomiting, particularly during the initial stages of treatment. In addition, Lin et al. [65] demonstrated that *CYP1A2* SNPs, such as rs4646425, rs2472304, and rs2470890, are associated with a slower response to paroxetine treatment. Furthermore, the study of the linkage between *CYP1A2* polymorphisms and the response to venlafaxine suggested that the rs2470890 polymorphism might be related to venlafaxine treatment remission [66].

#### 2.1.2. P-glycoprotein

P-glycoprotein, commonly referred to as P-gp, is a membrane transporter within the ATP-binding cassette (ABC) transporter family. It is encoded by the *ABCB1* or *MDR1* gene, located on chromosome 7. P-gp functions as an efflux pump, contributing to drug absorption, distribution, and elimination [12]. P-gp has been found in various tissues throughout the body, including the liver, kidneys, intestines, and the blood-brain barrier. At the blood-brain barrier, P-gp can limit the entry of some drugs into the brain, which can affect their efficacy. The expression and activity of P-gp can vary widely between individuals, contributing to differences in drug responses and drug interactions [18]. Genetic factors play a role in determining the level of P-gp expression in different tissues and individuals. Certain genetic variants of the *ABCB1* gene have been associated with altered P-gp expression and activity, which can affect the pharmacokinetics and efficacy of drugs that are substrates for P-gp [67]. P-gp has broad substrate specificity, including antidepressants such as escitalopram, fluvoxamine, paroxetine, amitriptyline, and imipramine. Therefore, when P-gp is absent or not functioning properly, these drugs can accumulate in the body, resulting in higher concentrations and potentially an increased risk of adverse effects. However, newer antidepressants (levomilnacipran, vortioxetine, and vilazodone) have been proven as poor P-gp substrates [18].

There has been interest in studying the relationship between genetic variations in the *MDR1*/*ABCB1* gene and antidepressant treatment outcomes [68]. Several genetic variants of the *MDR1*/*ABCB1* gene have been associated with altered P-gp activity. The three most common *MDR1*/*ABCB1* variants, C3435T (rs1045642), C1236T (rs1128503), and G2677T (rs2032582), have been the subject of extensive research [68,69]. A recent study on experimental models suggested that the 2677G > T polymorphism in the *ABCB1* gene has been associated with altered P-gp function and increased brain penetration of P-gp substrates without affecting P-gp protein expression in the blood-brain barrier [70]. In addition, genetic variants of the *ABCB1* gene, including the rs2235040 and rs4148739 polymorphisms, may be associated with the onset of response to antidepressant medications rather than the response rate [8]. Furthermore, a significant link has been found between the *ABCB1* rs2235015 GG genotype and better response to antidepressant treatment [8,71]. Overall, while pharmacogenetic research on *MDR1*/*ABCB1* suggests the potential for improving outcomes of antidepressant treatment, further investigation is necessary to better comprehend the connection between genetic variations and treatment response, as well as to assess its clinical utility.

### 2.2. Pharmacodynamic Variability

#### 2.2.1. Monoamine Metabolic Enzymes

##### Tryptophan Hydroxylase

Tryptophan hydroxylase (TPH) is an enzyme that catalyzes the conversion of the amino acid tryptophan to 5-hydroxytryptophan, which is the first step in the synthesis of serotonin, a neurotransmitter involved in the regulation of mood, appetite, sleep, and stress response [72]. There are two isoforms of the TPH enzyme, TPH1 and TPH2, encoded by separate genes. TPH1 is primarily expressed in peripheral tissues, such as the pineal gland, skin, and gut, whereas TPH2 is mainly expressed in neurons in the central nervous system (CNS). The two isoforms have similar enzymatic activities but differ in regulation, tissue distribution, and developmental expression patterns [73]. Overall, while TPH1 may be less expressed in the brain than TPH2, it appears to play an important role in the regulation of mood and stress responses and may contribute to antidepressant effects [74]. Several variants of *TPH* genes associated with differences in serotonin production have been identified. Some of these variants have been linked to an increased risk of developing psychiatric disorders [75]. The modulation of *TPH* genes has been studied as a potential approach for the treatment of various psychiatric disorders, including depression, anxiety, and addiction [76,77]. However, several studies have demonstrated controversial results regarding *TPH* polymorphisms and response to SSRIs [21,75,78].

##### Monoamine Oxidases

Monoamine oxidases (MAOs) are a family of enzymes that play critical roles in the metabolism of monoamine neurotransmitters in the CNS [79]. There are two types of MAOs, MAO-A and MAO-B, which are encoded by separate genes. MAO-A and MAO-B are found in the CNS, particularly in neurons and astroglia. MAO-A is primarily responsible for the metabolism of several important monoamine neurotransmitters, including serotonin, norepinephrine, and dopamine. These neurotransmitters play a crucial role in the regulation of mood, behavior, and cognition [80]. Several genetic variants of the *MAOA* gene associated with differences in enzyme activity and monoamine metabolism have been identified. In addition, some of these variants have been linked to an increased risk of developing psychiatric disorders [80]. However, the relationship between the *MAOA* gene variants and psychiatric disorders remains unclear. Polymorphisms in *MAO* genes have also been studied for their potential role in response to antidepressant medications. Some studies have suggested that certain genetic variants in *MAO* genes may affect the metabolism of antidepressants, potentially leading to differences in drug efficacy and side effects [81,82,83]. For example, individuals with a specific variant of the *MAOA* gene had a better response to fluvoxamine compared to those without this variant [82]. In addition, a recent study reported that the *MAOA* rs979605 polymorphism might modulate the response to antidepressant therapy in a sex-specific manner [84].

##### Catechol-O-Methyltransferase

Catechol-O-methyltransferase (COMT) is an enzyme that plays a crucial role in the degradation of catecholamines such as dopamine, epinephrine, and norepinephrine. The *COMT* gene has several polymorphisms, and the most extensively studied is the rs4680 polymorphism, also known as Val158Met [85]. This SNP causes the substitution of valine (Val) for methionine (Met) at position 158 in the COMT enzyme. The Val allele is associated with higher COMT activity, whereas the Met allele leads to lower activity. Therefore, individuals with the Val/Val genotype tend to have the highest level of COMT activity, followed by Val/Met individuals with intermediate activity, and Met/Met with the lowest activity [86]. The *COMT* Val158Met polymorphism has been implicated in various psychiatric disorders, including depression, anxiety, and schizophrenia [85]. Furthermore, studies have linked the *COMT* Val158Met polymorphism with response the to SSRIs such as fluoxetine and paroxetine [87,88]. For instance, one study discovered that the Val/Met genotype significantly affected the response to fluvoxamine [87]. Additionally, it has been suggested that the effects of the *COMT* genotype on antidepressant responses may depend on other factors, such as the type of medication, severity and duration of depression, and other genetic and environmental factors [89]. However, on the contrary, Brunoni et al. [21] did not observe a significant association between *COMT* variants and responses to escitalopram treatment.

#### 2.2.2. Monoamine Transporters

##### Serotonin Transporter

The serotonin transporter (SERT) encoded by the *SLC6A4* gene plays a critical role in regulating serotonin neurotransmission in the brain. SERT is responsible for serotonin reuptake from the synaptic cleft, thereby regulating the amount of serotonin available to bind to serotonin receptors in the brain [90]. Several classes of antidepressant drugs, including SSRIs, SNRIs, and TCAs, target SERT and inhibit its activity [91].

The *SLC6A4* gene was the first gene genotyped as part of the Genome-Based Therapeutic Drugs for Depression (GENDEP), and several genetic variants have been identified [92]. The most studied is the variant of the serotonin transporter-linked polymorphic region (5-HTTLPR). The 5-HTTLPR is located in the promoter region of the *SLC6A4* gene and has two alleles: the short (S) and the long (L) allele. The S allele is associated with lower transcriptional efficiency and reduced expression of the SERT protein compared to the L allele. As a result, carriers of the S allele have been found to have lower serotonin reuptake efficiency, leading to higher serotonin levels. Therefore, individuals carrying the S/S genotype have been shown to have a poorer response to SSRI treatment and may experience more side effects compared to those with the L/L or L/S genotypes [21,93]. Numerous studies have investigated the relationship between the 5-HTTLPR polymorphism and response to SSRIs [94,95,96,97]. A recent case report suggested that the S/S genotype resulted in ineffective fluoxetine treatment and may have caused exacerbation of depression [98]. Maron et al. [99] investigated the association between 5-HTTLPR polymorphism and clinical response to escitalopram and found no significant association. However, they did observe a linkage between this polymorphism and a higher risk for adverse effects [99]. This finding is consistent with other studies that have reported an association between SSRIs side effects and 5-HTTLPR polymorphism [100,101,102]. Therefore, the 5-HTTLPR polymorphism may be a valuable marker for predicting antidepressant responses and tolerability in individuals with psychiatric disorders. In particular, genotyping for the 5-HTTLPR variant may help identify patients who are less likely to respond to SSRIs and may benefit from alternative treatment strategies, such as SNRIs or TCAs. Additionally, several studies have reported an association between the 5-HTTLPR S/S genotype and suicidal behavior [102,103,104], although other risk factors, such as life stressors, psychiatric disorders, and substance use, must be considered.

##### Norepinephrine Transporter

The norepinephrine transporter (NET) is a protein encoded by the *SLC6A2* gene and plays an important role in the reuptake of norepinephrine (NE) [105]. TCAs, such as desipramine and imipramine, work by inhibiting the reuptake of NE by the NET, which increases the levels of NE in the synapse and enhances its effects on target neurons. Although antidepressants, such as SSRIs, have largely replaced TCAs, they are still used in some cases where other treatments have been ineffective. Several genetic variants have been identified in the human *SLC6A2* gene, which have functional consequences and can affect the efficacy of antidepressant drugs [105]. A recent study found an association between certain *SLC6A2* polymorphisms and antidepressant response variability [106]. Although, the role of *SLC6A2* variants in antidepressant responses is still being studied, current evidence suggests that these associations are complex and not fully understood.

##### Dopamine Transporter

The dopamine transporter (DAT), encoded by the *SLC6A3* gene, is a protein responsible for dopamine reuptake from the synaptic cleft into the presynaptic neuron. To date, the *SLC6A3* gene has been identified with at least 502 variations [107]. However, the relationship between *SLC6A3* polymorphisms and antidepressant responses is yet to be thoroughly researched. Nevertheless, some studies have suggested that specific *SLC6A3* polymorphisms may influence the response to antidepressant therapy [108,109]. For example, Kirchheiner et al. [110] suggested that the *SLC6A3* polymorphism increased the risk of a poorer and slower response to various antidepressants in individuals with the 9/10 and 9/9 genotypes compared to carriers of the 10/10 genotype.

#### 2.2.3. Monoamine Receptors

##### 5-HT_1A_ Receptor

The serotonin 1A (5-HT_1A_) receptor is a G protein-coupled receptor found in the CNS and peripheral tissues. It is widely distributed in various brain regions, including the hippocampus, hypothalamus, cortex, and amygdala. It plays a crucial role in regulating the release of neurotransmitters, particularly serotonin, and is a target for many psychoactive drugs, including antidepressants and anxiolytics [111]. The 5-HT_1A_ receptor is encoded by the *HTR1A* gene, and mutations in this gene have been linked to several neuropsychiatric disorders, including major depression, anxiety disorders, and autism spectrum disorder [112]. The role of *HTR1A* polymorphisms in response to antidepressant therapy has also been investigated; however, a few recent studies found no significant associations [113,114,115]. For instance, Scutt et al. [116] reported no association between *5HT1A* polymorphism and increased risk of SSRIs adverse effects. On the other hand, other studies have reported different findings [117,118]. Villafuerte et al. [117] suggested that the G allele of *HTR1A* rs1364043 polymorphism might predict citalopram response in depressed patients. This study showed that individuals homozygous for the G allele of the *HTR1A* rs1364043 polymorphism responded better to citalopram therapy. In patients with major depressive disorder, Kato et al. [118] found an association between *HTR1A* rs1364043 polymorphism and a better response to antidepressant treatment.

##### 5-HT_2A_ Receptor

The *HTR2A* gene, coding for the 5-HT_2A_ receptor, contains several polymorphisms, some of which have been linked to altered receptor function or expression. However, the *HTR2A* rs6311 and rs6313 polymorphisms have been researched the most, especially their association with response to antidepressant therapy [119]. Recent studies have shown that carriers of the *HTR2A* rs3803189 and rs7997012 polymorphisms responded better to SSRIs [120,121]. On the other hand, a number of other studies reported no linkage between different *HTR2A* polymorphisms and a patient’s response to antidepressant treatment [21,97,113,119,122]. Several studies have also investigated the relationship between different *HTR2A* SNPs and the tolerability of antidepressant therapy. According to their findings, specific *HTR2A* polymorphisms, such as rs7997012 and rs6314, may be associated with a reduced risk of adverse effects of antidepressant drugs [123,124].

##### Dopamine Receptors

Central dopamine receptors can be classified into two major families based on their structural and functional similarities: D1-like (D_1_ and D_5_ receptors) and D2-like receptors (D_2_, D_3_, and D_4_ receptors). Dopamine D_2_ receptors, encoded by the *DRD2* gene, are among the most intensively researched receptors in depressive disorders. Several studies have pointed out the possibility that *DRD2* polymorphisms play an important role in depressive disorder and response to antidepressant therapy [125,126]; however, other authors found no significant association [127,128]. On the other hand, Wang et al. [125] suggested that *DRD2* rs1076562, rs2440390, and rs2734833 polymorphisms are associated with the onset time of the antidepressant response. In addition, according to Perlis et al. [126], *DRD2* rs4245147 polymorphism has been linked to a better lamotrigine response in a group of patients with bipolar depression.

## 3. Pharmacogenetics of Anxiolytics

In comparison to antidepressants, pharmacogenetic research on anxiolytics is far less prevalent. This is due in part to the fact that benzodiazepines (BZDs), as a prototype of anxiolytic drugs, are administered for shorter periods. Some genes that have been studied regarding BZDs include those encoding drug-metabolizing enzymes, drug transporters, and drug targets [129]. Certain gene polymorphisms that can alter BZDs pharmacokinetics and pharmacodynamics are reviewed in the following sections.

### 3.1. Pharmacokinetic Variability

#### 3.1.1. UGT2B15 Enzyme

UGT2B15 is an enzyme essential for the metabolism of many drugs, including anxiolytics. It belongs to the uridine diphosphate glucuronosyltransferase (UGT) family of enzymes, which catalyze the conjugation of lipophilic compounds with glucuronic acid, thereby facilitating their elimination from the body [130]. UGT2B15 is primarily expressed in the liver and is encoded by the *UGT2B15* gene. Genetic polymorphisms in the *UGT2B15* gene can alter enzyme activity, leading to inter-individual variability in anxiolytic drug metabolism and response. For example, individuals with the UGT2B15*2 allele may have reduced UGT2B15 activity, resulting in slower drug metabolism and a potentially increased risk of adverse effects [131].

The UGT2B15*2 variant is one of the most studied genetic polymorphisms in the *UGT2B15* gene. This is due to a single nucleotide substitution (G > T) in the coding region of the gene, resulting in an amino acid change from aspartic acid (D) to tyrosine (Y) at position 85. This modification could alter the enzyme’s activity and therefore affect the metabolism of drugs that are substrates of UGT2B15 [132]. Chung et al. [133] were the first to demonstrate the effect of the *UGT2B15* genotype on the pharmacokinetics of lorazepam in humans. Their study showed that homozygous carriers of the UGT2B15*2 variant had lower systemic clearance and metabolic activity of lorazepam in comparison to individuals without this variant. Since then, several other studies have also examined the association between the *UGT2B15* genotype and the pharmacokinetics of lorazepam and other BZDs [132,134]. Mijdervik et al. [132] reported a linkage between the *UGT2B15* genotype and the efficacy of lorazepam in reducing postoperative anxiety. Interestingly, the effects of the UGT2B15*2 variant on the response to lorazepam premedication appeared to differ between male and female patients. Specifically, whereas homozygous male carriers of the UGT2B15*2 variant had a lesser reduction in anxiety compared to placebo, female patients with the same genotype experienced greater anxiety reduction due to lorazepam premedication [132]. However, Jackson et al. [134] suggested that the greater clinical effect in females compared to males after a single dose of lorazepam is unlikely to be due to differences in pharmacokinetics. Instead, they proposed that differences in endogenous levels of neurosteroid hormones between males and females may play a role.

Additionally, the UGT2B15*2 variant has also been shown to affect the metabolism of oxazepam. Several studies have shown that the UGT2B15*2 variant is associated with reduced oxazepam glucuronidation in human liver microsomes and decreased oxazepam clearance [131,135,136,137]. A decrease in oxazepam metabolism could potentially affect the metabolism of other BZDs, where oxazepam is an active metabolite, including chlordiazepoxide, clorazepate, diazepam, and temazepam [138]. This could lead to a potential increase in the adverse effects of these drugs, such as sedation, cognitive impairment, and impaired motor coordination [139]. Therefore, patients carrying the UGT2B15*2 variant may require lower doses of these BZDs in order to achieve the same therapeutic effect and minimize adverse effects.

#### 3.1.2. Cytochrome P450 Enzymes and P-glycoprotein

As previously discussed, the metabolism of many drugs, including anxiolytics, involves numerous CYP enzymes. Anxiolytic diazepam, for instance, is primarily metabolized by the CYP2C19 enzyme. Other CYP enzymes involved in diazepam metabolism include CYP2C9, CYP2B6, CYP3A4, and CYP3A5 [140]. The association between *CYPC19* and *CYP2B6* phenotypes and the safety of diazepam was the subject of a recent study by Zubair et al. [141]. This was the first study that demonstrated a significant association between the *CYP2B6* phenotype and the pharmacokinetics of diazepam. Their findings suggested that a dose reduction might be necessary for CYP2C19 and/or CYP2B6 PMs to avoid adverse drug reactions, such as dependence on and tolerance to BZDs. The *CYP2C19* gene has various genetic variants, some of which have the potential to influence CYPC19 enzyme activity. For example, the CYP2C19*17 allele is associated with increased enzyme activity, whereas nonfunctional variants, such as CYP2C19*2 and *3, have little or no **enzyme** function [140]. As stated before, these *CYP2C19* polymorphisms can result in altered rates of diazepam metabolism, leading to reduced drug effectiveness or potentially increased risk of adverse effects. Moreover, several recent studies have examined the effects of *CYP2C19* polymorphisms, particularly CYP2C19*17 and CYP2C19*2 variants, on the efficacy and safety of diazepam in individuals with alcohol withdrawal syndrome [142,143,144]. According to their findings, individuals with reduced CYP2C19 activity, such as carriers of the CYP2C19*2 allele, may be at an increased risk of adverse effects due to slower metabolism and potential drug accumulation. On the contrary, individuals with increased CYP2C19 activity, such as carriers of the CYP2C19*17 allele, may experience reduced efficacy with standard doses of diazepam due to faster metabolism and potentially lower plasma drug concentrations [142,143,144]. These findings are consistent with previous studies that investigated the effects of *CYP2C19* polymorphisms on diazepam metabolism and treatment outcomes in patients with alcohol withdrawal syndrome [145,146].

Similarly, the CYP2C19*17 allele may also be responsible for the UM phenotype of clobazam [147]. As a result, higher doses of clobazam may be required to achieve the same therapeutic effect. In contrast, CYP2C19*2 and CYP2C19*3 variants result in a nonfunctional or partially functional CYP2C19 enzyme. Individuals who are homozygous for CYP2C19*2 and CYP2C19*3 variants may have a PM phenotype with an increased risk of adverse effects and toxicity [140,148]. Two case reports have shown an association between the CYP2C19*2 variant and an increased risk of adverse effects in clobazam-treated patients [149,150]. As previously mentioned, the clearance of benzodiazepines, such as diazepam and clobazam, may be reduced in carriers of *CYP2C19* nonfunctional variants. However, BZDs have a wide therapeutic window, indicating that many individuals with this variant can still tolerate standard doses without experiencing substantial adverse effects [151]. Moreover, a recent study found an association between *CYP3A5* polymorphism and the metabolism of midazolam [152]. Specifically, carriers of the *CYP3A5* rs776746 T allele had decreased plasma concentrations of midazolam in comparison to individuals with the C allele and may require higher doses of midazolam to achieve a sedative effect [152]. The *CYP3A5* polymorphism, particularly the CYP3A5*3 variant, has also been associated with the altered metabolism of alprazolam [153]. Park et al. [154] have shown that homozygous carriers of the CYP3A5*3 allele might have a slower metabolism of alprazolam, resulting in higher plasma concentrations.

Regarding the CYP3A4 enzyme, the CYP3A4*22 gene variant has been frequently described in the literature [155]. Individuals who carry the CYP3A4*22 allele may have reduced CYP3A4 enzyme activity and slower drug metabolism [156]. Although, several studies have suggested that *CYP3A4* polymorphisms may be associated with differences in response to BZDs, the evidence is not yet conclusive [157,158]. In addition, there are currently no established pharmacogenetic biomarkers for BZDs such as bromazepam and lormetazepam [159]. As in the case of antidepressants, BZDs, such as midazolam, are also substrates for P-gp [160]. Several studies have investigated the potential role of P-gp polymorphisms in the response to midazolam [161,162]. However, these findings are inconsistent and sometimes contradictory. Specifically, whereas Park et al. [161] demonstrated that the polymorphism in the *MDR1*/*ABCB1* gene was linked to an increased midazolam concentration and higher sedation degree, Byon et al. [162] found no significant association.

### 3.2. Pharmacodynamic Variability

The pharmacodynamics of anxiolytic drugs are yet another target of pharmacogenetic studies investigating the treatment of various conditions [159]. It is well known that BZDs enhance the inhibitory effects of γ-aminobutyric acid (GABA) through the allosteric modulation of GABA type A (GABA_A_) receptors, resulting in anxiolytic, sedative, hypnotic, anticonvulsant, and myorelaxant effects [163]. The majority of GABA_A_ receptors are pentameric complexes composed of two α subunits, two β subunits, and one γ or δ subunit [164]. Depending on the GABA_A_ receptor subtype, the benzodiazepine effects can differ. The sedative effects of BZDs are primarily mediated by the α_1_ subunit of GABA_A_ receptors, whereas the α_2_ and α_3_ subunits are responsible for the anxiolytic effects of BZDs. Moreover, the α_5_ subunit mediates the amnestic properties of BDZs [165].

A study by Kelly et al. [166] showed that certain polymorphisms in the GABA_A_ receptor α_5_ subunit might result in a conformational change of the receptor, altering the GABA binding site. Several animal models with genetic mutations affecting specific subunits of the GABA_A_ receptor complex have been developed [167,168]. For example, one study found that mice lacking the γ_2_ subunit showed greatly reduced BZDs sensitivity, including the loss of sedative, anxiolytic, and anticonvulsant effects [169]. On the other hand, Chandra et al. [170] demonstrated that mice without the expression of γ_2_ exhibited increased anxiety-like behaviors but did not show significant differences in the hypnotic response to BZDs. In addition, mice with a knock-in F77I mutation in the *GABRG2* gene, coding for the GABA_A_ receptor γ_2_ subunit, were found to have reduced sensitivity to the hypnotic effects of zolpidem [171].

Polymorphisms in the *GABRA2* gene, encoding the α_2_ subunit of the GABA_A_ receptor, have been associated with substance abuse behaviors [172,173,174,175]. Another study investigated the connection between polymorphisms in the *GABRA1* gene and the response to zolpidem in patients with insomnia. The *GABRA1* A15G variant has been identified as a polymorphism associated with complex sleep behaviors and amnesia [176]. Choi et al. [177] found that among five known SNPs in the *GABRA1* gene, the rs4263535 polymorphism was associated with deeper sedation in response to intravenous midazolam. Moreover, Bowser et al. [178] discovered that individuals with epilepsy who carried the R43Q mutation in the *GABRG2* gene had reduced sensitivity to BZDs. Similarly, a recent study reported a possible association between two polymorphisms in *GABRA2* and *GABRA5* genes and drug-resistant epilepsy [179]. Overall, research assessing the pharmacodynamic variability of anxiolytic drugs remains a relatively new field. Additional research is necessary to fully understand how genetic variations affect anxiolytic drug responses.

## 4. Pharmacoepigenetics of Antidepressants and Anxiolytics

An increasing number of both preclinical and clinical findings suggest that epigenetic changes might be useful for the prediction of treatment response. DNA methylation, one of the most investigated epigenetic modifications, has been shown to play a role in drug responses, including responses to antidepressants and anxiolytics [180,181]. For instance, Takeuchi et al. [182] found an association between DNA methylation at several CpG sites in specific genes and the therapeutic response to paroxetine in patients with major depressive disorder. Moreover, methylation at several CpG sites within the interleukin-11 (*IL11*) promoter was found to be predictive of escitalopram or nortriptyline treatment response [183].

Several studies have also investigated the potential effects of epigenetic regulation of the brain-derived neurotrophic factor (*BDNF*) gene [184,185,186] on antidepressant treatment responses. For example, Wang et al. [186] suggested that *BDNF* DNA hypomethylation leads to an impaired response to escitalopram. In addition, lower methylation levels at CpG sites within the *BDNF* promoter were associated with an antidepressant response and decreased BDNF plasma levels after one week of treatment [187]. DNA methylation of *BDNF* seems to be epigenetically regulated by FK506 Binding Protein 5 (FKBP5), according to the antidepressant response [188]. FKBP5 is a cochaperone protein of the glucocorticoid receptor complex involved in intracellular glucocorticoid signaling and the stress response [189]. These findings are not surprising, since glucocorticoid secretion in response to stress, as well as genes involved in the glucocorticoid signaling pathway, are proposed to play an important role in shaping the epigenetic landscape [190]. Moreover, there is strong evidence that antidepressants exert their effects by modulating the glucocorticoid receptor; therefore, epigenetic modifications of the glucocorticoid receptor could be of considerable importance for therapeutic efficacy [191]. In the context of stress response, in addition to *FKBP5* and *BDNF*, other extensively studied epigenetically altered genes include the *NR3C1* gene coding for glucocorticoid receptor, *CRH* gene coding for corticotropin-releasing hormone, and *MAOA* and *SLC6A4* genes. Specifically, the study of Elliott et al. (2010) demonstrated the demethylation of *CRH* gene, involved in the brain stress response, in mice exposed to chronic social stress and attenuation of *CRH* promoter methylation levels with the antidepressant imipramine [192]. Moreover, hypomethylation in the promoter region of the *SLC6A4* gene has been predictive of impaired response to SSRIs [193,194,195]. On the other hand, investigations into the relationship between DNA methylation in the *MAOA* gene and the response to antidepressant drugs have produced conflicting results, with some studies reporting an association between *MAOA* DNA methylation and antidepressant response [196], while others observed no significant associations [197,198].

Various studies have implicated histone modifications in stress and depressive phenotypes as well as in antidepressant treatment. As in the case of DNA methylation, the *BDNF* gene has also been investigated regarding histone modifications. For instance, in mice, chronic social defeat stress induced lasting downregulation of *BDNF* transcripts III and IV and increased repressive histone methylation at their corresponding promoters in the hippocampus [199]. However, chronic imipramine treatment increased histone acetylation at the *BDNF* P3 and P4 promoters by selectively downregulating histone deacetylase (Hdac) [199]. In addition, Chen et al. investigated the effects of antidepressant drugs on the human postmortem prefrontal cortex and reported increased expression of *BDNF IV*, which was associated with a decrease in histone H3 lysine 27 (H3K27) methylation at the promoter region [200]. Moreover, a significant decrease in the peripheral blood H3K27 methylation levels at the promoter IV of the *BDNF* gene and a concomitant increase in *BDNF* mRNA expression were found in citalopram responders in comparison to nonresponders [185]. These findings suggest that *BDNF* promoter H3K27 methylation levels could serve as a potential biomarker for citalopram response in depressive patients.

Noncoding RNAs have also been investigated in relation to antidepressant responses [201,202]. Several miRNAs, such as miR-1202, miR124, miR-135a, miR-145, and miR-20b, have been strongly associated with antidepressant responses [203]. In addition, miR-146a5p, miR-146b-5p, miR-425-3p, and miR-24-3p expression levels decreased during antidepressant therapy and are therefore suggested as potential mediator biomarkers of antidepressant response [204]. In contrast to antidepressants, pharmacoepigenetic studies on anxiolytic drugs are still relatively limited. However, some evidence suggests that epigenetic modifications may also play a role in the drug response and efficacy of anxiolytics [205,206,207].

## 5. Conclusions

Pharmacogenetics can potentially optimize the treatment of neuropsychiatric disorders via the prediction of clinical outcomes of antidepressant and anxiolytic drug administration, including therapy response and development of drug side effects, without the need for conventional “trial-and-error” approaches. The PREPARE study, which covered a large number of drugs, demonstrated that genotype-guided treatment using a 12-gene pharmacogenetic panel significantly reduced the incidence of clinically relevant adverse drug reactions [208]. These results show the large-scale feasibility and clinical usefulness of implementing of a panel-based pharmacogenetic testing strategy to make drug therapy increasingly safe [208].

**Figure 2:**
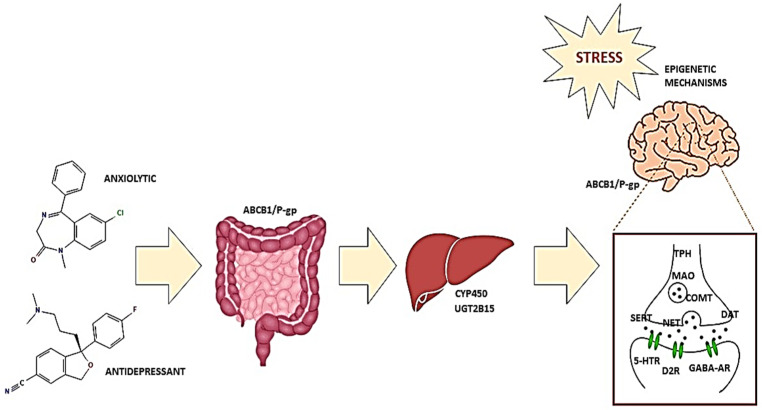
Pharmacogenetics can potentially optimize the treatment of neuropsychiatric disorders via the prediction of clinical outcomes of antidepressant and anxiolytic drug administration based on patients’ genetic variations affecting metabolizing cytochrome CYP450 and UGT enzymes, P-glycoprotein ABC transporter, as well as monoamine and GABA metabolic enzymes, transporters, and receptors. Some of these gene candidates, as well as many others, may be epigenetically altered by stress. Pharmacoepigenetics can help make clinically relevant decisions regarding antidepressant and anxiolytic therapy by investigating how epigenetic mechanisms influence drug responses.

As shown in Figure 2, for antidepressant and anxiolytic therapy, the most promising pharmacogenetic candidates are genetic polymorphisms affecting metabolizing cytochrome CYP450 and UGT enzymes, P-glycoprotein ABC transporter, as well as monoamine and GABA metabolic enzymes, transporters, and receptors. Some of these gene candidates, as well as many others, have been shown to be epigenetically altered in response to stress, suggesting the potential of epigenetic profiles for making clinically relevant decisions regarding antidepressant and anxiolytic therapy.

Although pharmacogenetic research has revealed that more efficient and safer treatment with antidepressants and anxiolytics can be achieved through genotype-guided decisions, further well-designed trials are needed to overcome various methodological limitations and confirm the current pharmacogenetic findings. The other main constraints of pharmacogenetic studies targeting both pharmacokinetic and pharmacodynamic variability include the high cost of molecular testing, their relative inaccessibility, and the complexity of result interpretation. Nevertheless, with more evidence on the clinical and economic benefits, improved clinical guidelines, lower costs, and shorter delivery times, pharmacogenetics as a key part of personalized medicine may become a routine intervention in neuropsychiatric clinical practice. On the other hand, the pharmacoepigenetic field is still relatively new compared to pharmacogenetic research. Therefore, future research advances on the relationship between epigenetic modifications and drug responses are needed in order to personalize antidepressant and anxiolytic treatment.

## Data Availability

Not applicable.
